# Antifeedant Potential of Geranylacetone and Nerylacetone and Their Epoxy-Derivatives against *Myzus persicae* (Sulz.)

**DOI:** 10.3390/molecules27248871

**Published:** 2022-12-13

**Authors:** Anna Wróblewska-Kurdyk, Katarzyna Dancewicz, Anna Gliszczyńska, Beata Gabryś

**Affiliations:** 1 Department of Botany and Ecology, University of Zielona Góra, Szafrana 1, 65-516 Zielona Góra, Poland; 2 Department of Food Chemistry and Biocatalysis, Wrocław University of Environmental and Life Sciences, Norwida 27, 50-375 Wrocław, Poland

**Keywords:** antifeedants, green peach aphid, aphid probing, EPG, geranylacetone, epoxygeranylacetone, nerylacetone, epoxynerylacetone

## Abstract

Geranylacetone and nerylacetone are natural sesquiterpenoids, which play various roles in plant-insect interactions, including the deterrent and repellent effects on herbivores. The structural modifications of natural compounds often change their biological activities. The aim of the study was to evaluate the effect of geranylacetone, nerylacetone and their epoxy-derivatives on the probing and settling behavior of *Myzus persicae* (Sulz.) (Hemiptera: Aphididae). The no-choice test using the Electrical Penetration Graph (EPG) technique showed that the probes before the first phloem phase were usually shorter than 3 min, which means that they were terminated within the epidermis and/or outer layers of mesophyll. This resulted in a tendency to delay the initiation of the phloem phase in aphids, which reflects a weak preingestive deterrent activity of the studied compounds at the level of non-vascular tissues. Most *M. persicae* showed bouts of sustained phloem sap ingestion. However, the 24-h free-choice test demonstrated that aphids did not settle on the leaves treated with geranylacetone, nerylacetone, and their epoxy-derivatives. The refusal to settle after the consumption of phloem sap on treated plants indicated that the studied compounds had postingestive deterrent activity. The epoxidation of geranylacetone and nerylacetone did not evoke significant changes in their activity profiles.

## 1. Introduction

Aphids (Hemiptera: Aphididae) are one of the most economically important groups of insect pests in agriculture in temperate climates [1]. The annual world crop damage caused by aphids is estimated to cause tens of millions to billions of US$ of yield loss [2]. Often, as large colonies, aphids may damage plant vitality directly by ingesting their phloem sap and indirectly through the production of honeydew which covers the leaves and fruits, and most importantly, also by the transmission of plant pathogenic viruses [2,3]. The green peach aphid *Myzus persicae* (Sulz.) is an important insect pest of many crops worldwide both in the field and in the greenhouses [3,4]. The green peach aphid is a highly polyphagous species that has an extremely wide range of host plants belonging to more than 400 species of 50 plant families [5,6]. The great economic importance of *M. persicae* is due to its efficiency as a virus vector. It has been shown to be able to transmit considerably more than 100 plant viruses, both persistent and non-persistent viruses [7]. The green peach aphid is now resistant to several classes of insecticides, which is a consequence of the intensive use of various insect control products [8]. Therefore, an alternative method of aphid control is needed and the use of targeted chemicals that would repel aphids or deter their probing and feeding is one of the most promising approaches [9].

Globally, agricultural producers applied approximately 7.2 Mt of formulated products, including insecticides, in 2020 [10]. Insecticides are widely used to control insect pests, but their widespread use poses a threat to the environment and non-target organisms, ranging from beneficial soil microorganisms, to insects, fishes, and birds [11]. Therefore, the application of natural insecticides in agriculture has been increased as an alternative to synthetic insecticides [12]. In current plant protection, attention is increasingly being paid to the use of secondary plant metabolites, which in nature protect many plants from most insect herbivores [13,14]. The role of secondary metabolites in defense may involve repellent and antifeedant activities and also the inhibition of reproduction and other life processes [15,16]. Till now, the antifeedant activity of about 1000 plant compounds has been tested against insects significant in agriculture and forestry. Based on the known activity and chemical structure, the most active substances can be commercialized as natural plant protection products [17]. So far, the main insecticides based on plant allelochemicals present in the global biopesticide market are azadirachtin and pyrethrum [18,19]. Azadirachtin, a tetranortriterpenoid derived from the neem seed of the Indian neem tree (*Azadirachta indica* A. Juss (Meliaceae)) is the most successful botanical pesticide in agricultural use worldwide, being a powerful antifeedant against 413 insect species and at the same time non-toxic to biocontrol agents such as predators and parasitoids, and mammals [20,21]. Another substance discovered for insect control is the sesquiterpenoid polygodial which was successfully applied in the field against the bird cherry-oat aphid *Rhopalosiphum padi* (L.). It gave results similar to those obtained with broad-spectrum cypermethrin [22]. Among the plant products against phytophagous insects, essential oils are growing in importance because of their relative no toxicity to birds, fish, and mammals [23]. They have low persistence under field conditions, which makes them relatively safe for the environment [23,24]. The insecticidal activity of lavender, rosemary, and peppermint essential oils against *Aphis gossypii* Glover is broadly known [25]. In addition, the same oils and also orange oil significantly reduces the progeny of cotton aphid *Aphis gossypii* Glover under laboratory testing conditions [26]. The main components of essential oils are lower terpenoids—mono- and sesqui-terpenoids—which are neurotoxic, unpalatable, or at least repellent to insect herbivores [27,28]. As such, these substances may be extremely potent feeding inhibitors to a number of insect species [13,29,30]. The repellent activity of citral and linalool to *M. persicae* was demonstrated by Gabryś et al. [31] while Halbert et al. [32] found that geraniol, farnesol, and β-citronellol have a strong repellent effect on *Rhopalosiphum maidis* (Fitch).

Geranylacetone and nerylacetone are natural sesquiterpenoid components of plant essential oils [33,34,35,36]. Geranylacetone is bactericidal and fungicidal [37]. Used against the higher organisms, geranylacetone had a deterrent activity to the bark beetle *Ips subelongatus* Motsch. (Coleoptera: Curculionidae) and a repellent activity to ticks *Rhipicephalus appendiculatus* Neum. (Ixodida: Ixodidae) [38,39]. Nerylacetone is an ingredient many essential oils obtained from, e.g., *Paulownia tomentosa* (Thunb.) (Paulowniaceae) and ginger *Zingiber officinale* Roscoe (Zingiberaceae), which have antibacterial activity [40,41]. Considering their broad activity, it is very likely that geranylacetone and nerylacetone may affect the behavior of the green peach aphid, especially modify its foraging activity. The effects of geranylacetone and nerylacetone on *M. persicae* have never been studied and remain unknown. We decided to carry out the present study to fill this gap. Moreover, knowing that structural modifications may affect the activity of the natural compounds, we also investigated the effects of related compounds, namely the epoxy-derivatives of geranylacetone and nerylacetone [42].

The main goal of the present study was to explore in detail the effects of geranylacetone and nerylacetone and their epoxy-derivatives application on *M. persicae* behavior, which could provide knowledge on the possibility of the use of these compounds in the sustainable control of this aphid species. The behavioral background of the deterrent effects of tested sesquiterpenoids was studied by direct observation of aphid settling (choice-test) and the monitoring of aphid stylet penetration in plant tissues using the Electrical Penetration Graph (EPG) technique (no-choice test). The EPG technique allows for the recording of the probing behavior of phytophagous insects with piercing-sucking mouthparts, therefore it is commonly used to study the interactions between Hemiptera and plants [43]. Electronic monitoring of the stylet penetration of plant tissues and the phloem sap ingestion by aphids provides valuable and accurate information concerning the nature and tissue localization of factors modifying the behavior of these insects [44,45,46]. This method is also used to study the mechanism of plant virus transmission by aphids and to detect natural resistance in wild and cultivated plant species and varieties [47,48,49]. In our study, we were particularly interested which phases of aphid probing in plant tissues were, if at all, the most strongly affected by the application of geranylacetone and nerylacetone, how the epoxidation of these molecules affected their activity, and, finally, what consequences this might have on plant infestation by *M. persicae*.

## 2. Results

### 2.1. Chemical Synthesis

**Figure 1:**
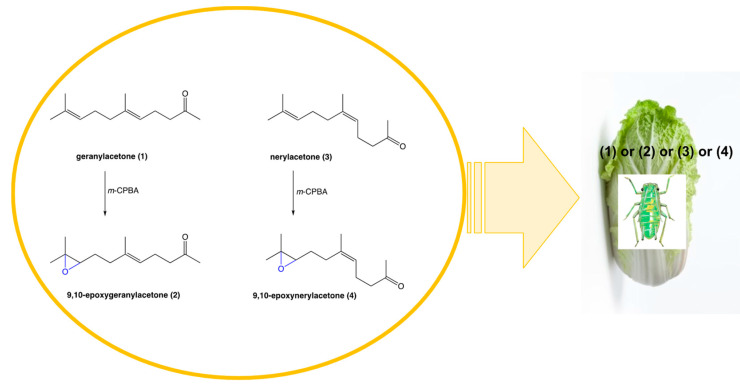
Chemical synthesis and application of 9,10-epoxygeranylacetone (**2**) and 9,10-epoxynerylacetone (**4**).

Racemic (±)-9,10-epoxygeranylacetone (**2**) and (±)-9,10-epoxynerylacetone (**4**) were obtained from geranylacetone (**1**) and nerylacetone (**3**)**,** respectively in the reaction of oxidation performed with *m*-CPBA in the anhydrous dichloromethane. Reactions were conducted at a low temperature of 0°C to protect the double bond between carbon atoms C-5 and C-6 and receive monopoxyderiveatives. Products were obtained in good 86% (for 9,10-epoxygeranylacetone (**2**)) and 72% (for 9,10-epoxynerylacetone (**4**)) yields (Figure 1) and their structures were confirmed based on their spectroscopic analysis. The presence of oxiran rings in the structures of (**2**) and (**4**) were confirmed by triplets at 2.66 and 2.51 ppm respectively from protons H-9 in the ^1^H NMR spectra. The evidence for the occurrence of the oxiran rings was provided also by ^13^C NMR where the signals from C-9 and C-10 were shifted to higher field δ = 64.12 and 58.34 in the spectrum of (**2**) and to δ = 64.00 and 58.33 in the spectrum of (**4**) in comparison to the spectra of substrates geranylacetone (**1**) and nerylacetone (**3**).

### 2.2. Aphid Settling (Choice-Test)

**Figure 2:**
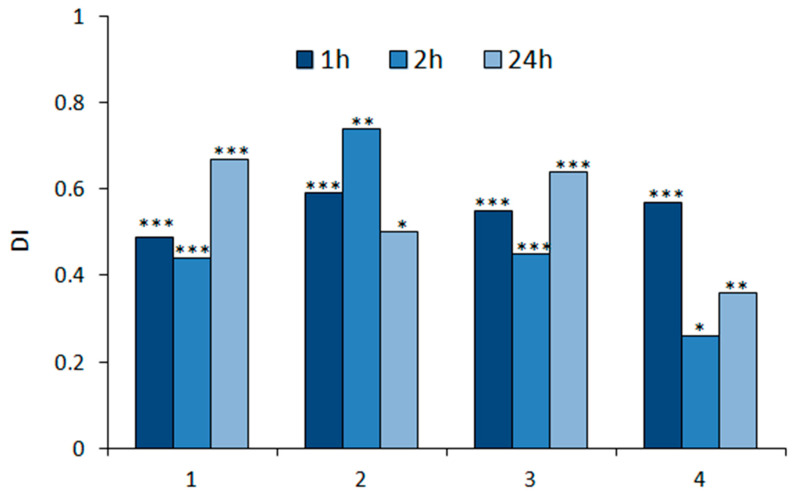
Settling success of *Myzus persicae* on *Brassica rapa* subsp. *pekinensis* exposed to geranylacetone (**1**), epoxygeranylacetone (**2**), nerylacetone (**3**), and epoxynerylacetone (**4**). The same plant species was used to maintain the aphid stock culture. The relative index of deterrence (DI) after 1, 2, and 24 h. Asterisks indicate statistically significant differences between the numbers of aphids on control and treated leaves according to the Student *t*-test: * *p* < 0.05, ** *p* < 0.01, *** *p* < 0.001.

The potency and durability of deterrent effects on *M. persicae* settling activity after exposure to all sesquiterpenoids tested were very high (Figure 2). The deterrent effects were manifested as soon as 1 h after the freely moving aphids were confronted with the treated leaves and lasted at least until the end of the experiment, which was 24 h (Table 1, Figure 2). Aphids settled mainly on control leaves (on average 63–86% of the aphids participating in the experiment) (Table 1).

### 2.3. Electronic Registration of Aphid Probing Behavior (No-Choice Test)

#### 2.3.1. General Aspects of Aphid Probing Behavior

**Figure 3:**
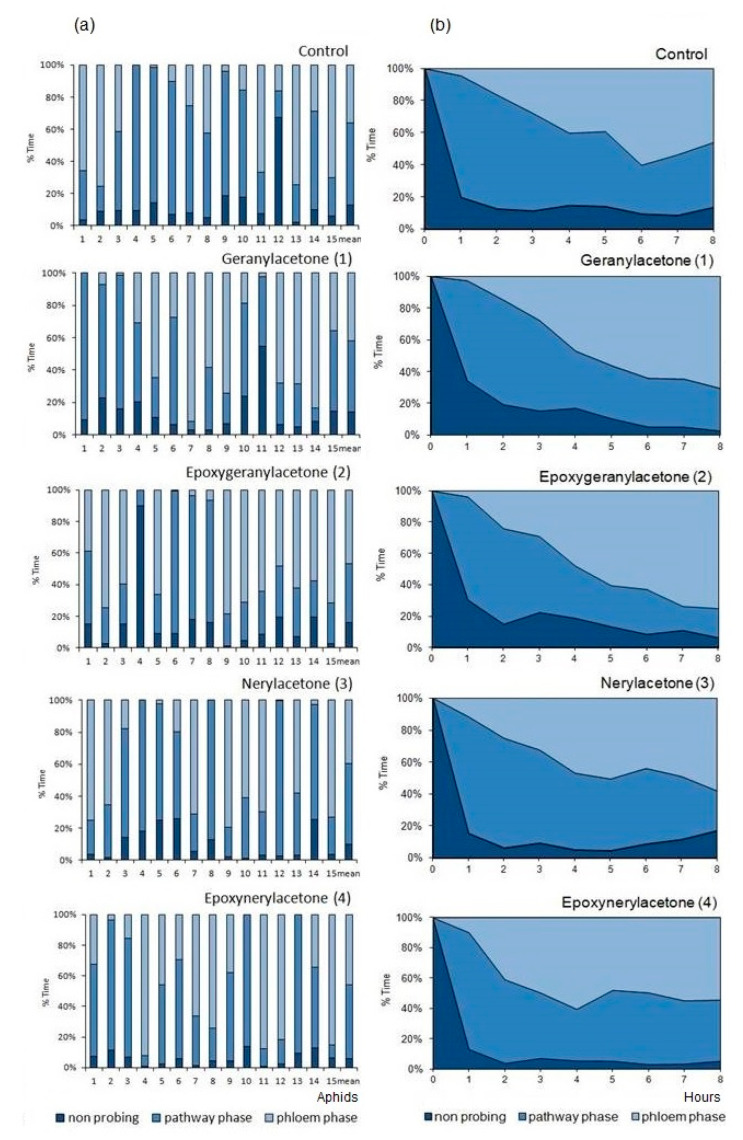
A general outline of the probing behavior of *Myzus persiace* on *Brassica rapa* subsp. *pekinensis* treated with sesquiterpenoids geranylacetone (**1**), epoxygeranylacetone (**2**), nerylacetone (**3**), and epoxynerylacetone (**4**)**.** (**a**) Individual variation in probing behavior of *M. persicae* on control and sesquiterpenoid-treated plants; numbers denote individual aphids. (**b**) Sequential changes in *M. persicae* probing behavior on control and sesquiterpenoid-treated plants; non-probing (aphid stylets outside the plant), pathway phase (probing in non-phloem tissues; waveforms A, B, C, F, and G); phloem phase (waveforms E1 and E2).

The foraging activities of *M. persicae* on control and sesquiterpenoid-treated plants consisted mainly of probing, i.e., active penetration of mouthparts’ stylets in plant tissues, which occupied 83% of the experimental time on epoxygeranylacetone (**2**)-treated plants to 93% on epoxynerylacetone (**4**)-treated plants (Table 2, Figure 3a). Probing activities comprised non-phloem and phloem probing phases (Table 2). The phloem phase, i.e., the salivation and the ingestion of phloem sap occupied 49% of the probing time on geranylacetone (**1**)-, 56% on epoxygeranylacetone (**2**)-, 44% on nerylacetone (**3**)-, and 49% on epoxynerylacetone (**4**)-treated plants, respectively, which was similar to the control where phloem phase embraced 63% of probing activities (Table 2, Figure 3a).

In the course of time, the proportion of time dedicated to individual phases of probing changed, but the trends were similar in all treatments, regardless of the compound that was applied to the aphid host plant. Non-probing and pathway activities predominated during the first hour of the experiment in all aphids on all plants (Figure 3b). Later, the proportion of the non-probing phase decreased while the proportion of non-phloem and phloem probing phases in aphid activities increased, and at the end of the 8 h experiment, the phloem phase was the main aphid activity on all plants (Figure 3b).

The probing activities of *M. persicae* were slightly enhanced on plants treated with epoxynerylacetone (**4**) in relation to the control (Table 2). The application of geranylacetone (**1**) and epoxygeranylacetone (**2**) caused a reduction in the total number of contacts with the sieve elements expressed as ‘E’ waves in the electropenetrogram in relation to control. On geranylacetone-(**1**) and epoxygeranylacetone (**2**)-treated plants, the frequencies of contacts with the sieve elements were 3 and 2.7 times lower than on the control, respectively (Table 2).

#### 2.3.2. Aphid Probing Behavior Prior to the First Phloem Phase (Non-Phloem Tissues)

**Figure 4:**
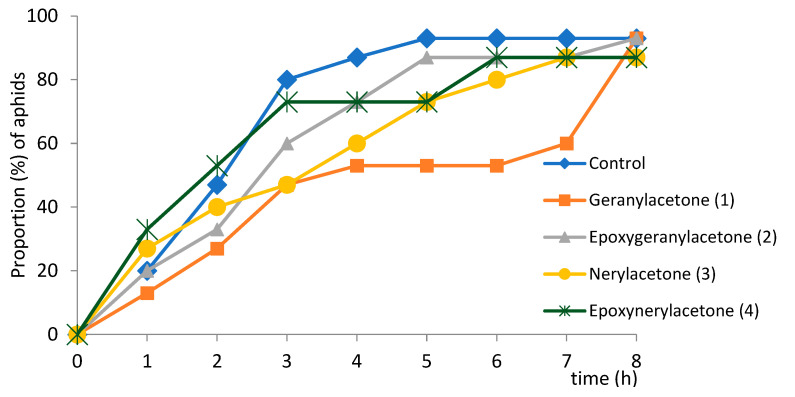
Trends in the cumulative proportion of *Myzus persicae* on *Brassica rapa* subsp. *pekinensis* that made contact with sieve elements during 8-h access to plants after exposure to geranylacetone (**1**), epoxygeranolacetone (**2**), nerylacetone (**3**), epoxynerylacetone (**4**).

On all treated plants, the main activity of aphids was probing in non-phloem tissues (52–69%). The majority (from 53% in aphids on epoxynerylacetone-treated plants to 71% on epoxygeranylacetone-terated plants) of probes that occurred before the first phloem phase were shorter than 3 min. (Table 2). Nearly all aphids started the phloem phase on all plants within the 8-h experiment (Figure 4). The average time to locate phloem vessels was from 2.1 (±2.5) h on epoxynerylacetone-treated (**4**) plants to 3.9 (±2.9) h on geranylacetone-treated (**1**) plants; on control, aphids needed 2.4 (±2.1) h to locate phloem vessels (Table 2, Figure 4). Although the differences in the time to reach the phloem phase were statistically not significant (Table 2), there was a certain variation among the studied aphid populations in terms of the proportion of aphids that started ingestion within a given time (Figure 4). On control and epoxynerylacetone-treated plants (**4**), the majority of aphids started the phloem phase within three hours after they had access to plants, which was similar to control, while it was four-five hours for the same proportion of aphids on epoxygeranylacetone-treated plants (**2**), seven hours on nerylacetone-treated (**3**) and eight hours on geranylacetone-treated plants (**1**) (Figure 4).

#### 2.3.3. Aphid Probing Behavior during Phloem Phase (Phloem Tissues)

The proportion of the phloem phase during probing and the proportion of salivation in the phloem phase were similar to the control. Furthermore, the application of geranylacetone (**1**) and epoxynerylacetone (**4**) caused a significant increase in the mean duration of an individual period of sap ingestion, respectively 1.4 h and 1.9 h (Table 2).

#### 2.3.4. ‘Exploratory Cell Punctures’ in Non-Phloem Tissues

The proportion of probes that included exploratory ‘short’ and ‘long’ cell punctures (pd-S and pd-L, respectively) was by 23% lower on epoxygeranylacetone (**2**)-treated plants than on control. The total number of pd-S exploratory cell punctures was 1.5 times lower and the cumulative durations of subphases II-3 in all pd-S were 1.5 times shorter on epoxygeranylacetone (**2**)-treated plants in relation to control. The mean duration of a single pd-S per probe was lower on geranylacetone (**1**) and nerylacetone (**3**)-treated plants. The cumulative durations of subphases II-1 and II-2 in all pd-S were significantly shorter on geranylacetone (**1**) and nerylacetone (**3**)-treated plants in relation to control. In pd-L exploratory cell punctures, the only significant difference in relation to control concerned the mean duration of a single pd-L per probe, which was lower on nerylacetone (**3**)-treated plants than in aphids on control plants (Table 3).

## 3. Discussion

In the present study, the deterrent activity of nerylacetone, geranylacetone, and their epoxy-derivatives was examined in two independent complementary experiments, one involving freely moving aphids (aphid settling in a choice-test), the other involving tethered aphids (aphid probing behavior in the EPG no-choice test). The aim of the choice test was to show aphid preferences for the food source and the aim of the no-choice test was to explain the background of the observed preferences. The no-choice test, i.e., the 8 h continuous monitoring of aphid behavior allows the analysis of probing during the pre-ingestive (within non-phloem tissues before the first phloem phase) phase separately from the ingestive (within the phloem) phase of probing [42]. The parameters derived from the electronic registration are good indicators of plant suitability or interference of probing by chemical or physical factors, including the exogenously applied chemicals, in individual plant tissues and the association of this activity to particular phases of aphid probing [42,44,45,47,50,51,52,53]. When xenobiotics are applied exogenously, the aphids may act as sensors that indicate the transfer of these compounds from the epidermis into internal plant tissues [44,50].

The results of EPG monitoring in the present study showed weak direct effects of nerylacetone, geranylacetone and their epoxy-derivatives on the behavior of *M. persicae* during probing. In some cases, the total duration of probing was longer (**4**), the number of phloem phases was lower (**1**, **2**) and the mean duration of sap ingestion was longer (**1**, **4**) as compared to the control values. These changes may be evidence of relatively weak deterrent factors located in non-phloem and phloem tissues [45]. Phloem sap ingestion was the main activity during probing and the bouts of sap ingestion were long and rarely interrupted which means that the phloem sap might have had an attractive taste or had other characteristics that promoted feeding. Similar behavior of aphids is observed on plant cultivars that vary in susceptibility to aphid infestation [47,48]. On highly acceptable cultivars of *Lupinus luteus* L., the total and mean duration of probing, time to reach the phloem phase, and the duration of the first phloem phase were comparable to those in the pea aphids *Acyrthosiphon pisum* Harris on control plant *Pisum sativum* L., whereas on unpalatable cultivars, the phloem phase occurred rarely, and when it did, it was short and consisted mainly of watery salivation [48]. Such aphid probing behavior is characteristic of host plants that lack feeding deterrents [54,55]. Generally, deterrent factors are secondary metabolites, which are essential components of immunity in plants against harmful insects [56]. In the tissues of lupines that were poorly accepted or entirely not accepted by the pea aphid, the deterrent factors were mainly lupanine and its derivatives [48]. In the study by Kordan et al. [47], on highly susceptible rapeseed varieties, all studied *M. persicae* achieved phloem phase and all showed sap ingestion activity in a relatively short time and the duration of feeding was relatively high, which suggests the absence of antixenosis mechanisms in the phloem sap in these cultivars [57]. In studies concerning exogenously applied allelochemicals, rutin caused a delay in reaching sieve elements by *A. pisum* on *P*. *sativum* and deterred probing activities of *M. persicae* within non-phloem tissues on *B. rapa* subsp. *pekinensis*, daidzein caused a delay in reaching phloem vessels and limited sap ingestion in *A. pisum*, and damascone-derived dihydro-β-damascol, β-damascone acetate, δ-bromo-γ-lactone, and unsaturated γ-lactone—affected pre-phloem and phloem probing activities of *M. persicae* [44,58,59]. Likewise, the isprenoids camphene- and β-ionone caused an increase in the proportion of non-probing relative to other stylet activities, a decrease in the success rate in reaching sieve elements and feeding, and an increase in the proportion of salivation in the phloem phase in *M. persicae* [50].

Frequently, during the stylet pathway to the phloem, aphids puncture epidermal cells and ingest small samples of cytoplasm for gustatory purposes in the host plant recognition process [60,61]. In EPG recordings, brief intracellular punctures are identified as short (pd-S) and long (pd-L) potential drops (pd) [61,62,63,64,65]. Short potential drops (pd-S) have two pulses or less, whereas long potential drops (pd-L)—three pulses or more in the subphase II-3. Very often, long potential drops occur as the first intracellular punctures after the stylet insertion into the plant by the aphid [63]. Aphid behavior during brief probes (pd-S and pd-L) is related to the transmission of non-persistent and semi-persistent plant viruses [63,64,66,67]. Moreno et al. [64] showed that the success in the acquisition of semi-persistent virus *Cauliflower mosaic virus* (CaMV), acquired from non-phloem tissues was associated with a longer duration of intracellular punctures. The frequency of intracellular stylet punctures (potential drops) was associated with high effectiveness of transmission of potyviruses, *Potato virus Y* (PVY), and *Plum pox virus* (PPV) by *M. persicae* and the duration of long potential drops was positively correlated with a high transmission efficiency of *Cucumber mosaic virus* (CMV) by *A. gossypii* [63,68]. Moreno et al. [66] proved that the inoculation of non-persistent *Turnip mosaic potyvirus* (TuMV) occurs during subphase II-1 of the intracellular puncture, whereas inoculation of semi-persistent CaMV—during subphase II-2. The acquisition of a non-persistant virus occurs primarily during the last subphase (II-3) of intracellular stylet punctures [64]. The present study showed that the application of geranylacetone (**1**), epoxygeranylacetone (**2**), and nerylacetone (**3**) may potentially reduce the effectivity of non-persistent and semi-persistent virus transmission, evidenced by the lower frequency of pd-S (**2**) and reduced mean duration of a single pd-S (**1, 3**), total duration of subphase II-1 of pd-S (**1, 3**), total duration of subphase II-2 of pd-S (**1, 2, 3**), mean duration of a single pd-L (**3**). The application of epoxynerylacetone only reduced the frequency of pd-L during a single penetration. However, a study dedicated to virus transmission will be needed to precisely explore this possibility.

The biological activity of a given compound is species-specific and depends on its structural characteristics. Variations, such as the incorporation of functional groups or epoxidation can produce radical changes in activity [69]. In our previous studies, we determined that chemical modifications of naturally occurring compounds can evoke significant changes in their activity profiles, for example, stronger and more durable deterrent effects, shifts from attractant to deterrent properties, or vice versa [44,70,71]. One example of such changes is the introduction of a lactone moiety and a halogen atom into a piperitone molecule, which dramatically changed its biological activity. The piperitone-derived chlorolactones and bromolactones had a very strong deterrent effect on aphid settling in contrast to the original compound [70]. Lactamization of thujone resulted in more durable deterrent effects in aphid settling and revealed deterrent properties during probing in non-phloem tissues [71]. Chemical modifications of naturally occurring compounds can cause also the loss of the deterrent activity such as in the case of (+)-13-hydroxynootkatone which is a product of biotransformation of the (+)-nootkatone and proved to be completely inactive against *M. persicae* [71,72]. In the present study, the epoxidation of natural terpenoids geranylacetone (**1**) and nerylacetone (**3**) did not evoke significant changes in their activity profiles, similar to the research by Paprocka et al. [42] when the epoxydation process of the cis-jasmone molecule did not change the activity profile: epoxy-derivatives of geranylacetone (**1**) and nerylacetone (**3**) limited the settling of *M. persicae* on plants and limited the probing activity in non-phloem tissues. However, in our other studies, the epoxidation process changed the properties of the transformed starting compounds. The natural compound, trans, trans-farnesol appeared a very strong deterrent to *M. persicae*, but its epoxy-derivative was inactive [73]. (+)-Nootkatone limited aphid settling after 24 h exposure and (+)-11,12-epoxy-9α-hydroxynootkatone—after 1 h and 2 h [72]. Another example is hydroxyjasmone which showed weak but not significant deterrent properties while its epoxy-derivative, the epoxylactone was significantly highly attractant to *M. persicae* and this activity persisted for at least 24 h [74].

The choice test for freely moving aphids reveals the preferences of aphids during at least 24 h after exposure to the studied allelochemicals and indicates the possible postingestive activity of the applied substances, provided the aphids are able to feed upon phloem sap of the treated plants [75]. We demonstrated in the EPG experiments that almost all *M. persicae* were able to locate sieve elements and ingest phloem sap in a sustained way irrespective of the applied sesquiterpene. In the choice test, we showed that geranylacetone (**1**), nerylacetone (**3**), and their epoxy-derivatives (**2, 4**) acted as deterrents for at least 24 h. The free aphids refused to settle on all studied sesquiterpenoid-treated leaves, although they spent similar time on probing activities in non-phloem tissues and just as long on sustained feeding on phloem sap as on control untreated plants. The avoidance of the treated leaves during settling might have been the delayed effect of consuming the toxic sap from geranylacetone (**1**), nerylacetone (**3**), and its derivatives (**2, 4**)-treated leaves. This may suggest the postingestive effects of all studied sesquiterpenoids, probably due to metabolic reasons. Similar activity profiles of deterrent compounds that act during at least 24 h when aphids attempt to settle on plants but with no significant effect on aphid probing were observed also in our previous studies, e.g after application of chlorolactones derived from piperitone [70]. It is likely that similar reasons might have caused the observed phenomenon in the present study: the studied compounds were in all probability not deterrent in a gustatory manner but had a postingestive and delayed effect on aphid settling behavior. The potency of the deterrent effect increased in the course of the 24 h experiment. The posingestive effect was also observed after the application of two piperitone-derived δ-hydroxy-γ-lactones with the p-menthane system and a citral derivative with an α-methylenelactone moiety [76,77]. Similar effects on aphid behavior during the phloem phase were revealed when aphids were offered plants treated with farnesol. The freely moving aphids were reluctant to remain on (E,E)-farnesol-treated leaves for at least 24 h after exposure, but the feeding of *M. persicae* was disturbed by the application of this compound [78]. Nevertheless, the weak deterrent activity of pre-ingestive character of geranylacetone (**1**), nerylacetone (**3**), and their epoxy-derivatives (**2, 4**) can not be excluded as aphids were reluctant to continue probing before the first phloem phase: most probes were terminated within 3 minutes from the insertion of the stylets.

## 4. Materials and Methods

### 4.1. Compounds and Reagents

Geranylacetone (**1**), nerylacetone (**3**), and *m*-chloroperoxybenzoic (*m*-CPBA) acid were purchased from Aldrich. (±)-9,10-Epoxygeranylacetone (**2**) and (±)-9,10-epoxynerylacetone (**4**) were synthesized from **1** and **3** respectively in the reaction with *m*-CPBA. Dichloromethane was purchased from Merck.

### 4.2. Synthesis of Epoxyderivatives of Geranylacetone and Nerylacetone

To the solution of geranylacetone (**1**) or nerylacetone (**3**) (0.01 mol) dissolved in dichloromethane (30 mL), the solution of *m*-CPBA (0.01 mol) also in dichloromethane was added dropwise. The reaction mixture was stirred at 0 °C for 3 h. After this time the mixture was washed with Na_2_SO_3_, NaHCO_3_ and brine. The organic solution was dried over MgSO_4_ and the products were next purified using a chromatography column (silica gel, hexane:acetone, 10:1). (±)-9,10-Epoxygeranylacetone (**2**) and (±)-9,10-epoxynerylacetone (**4**) were obtained with 86 and 72% yield, respectively.

*(±)-9,10-Epoxygeranylacetone* (**2**): ^1^H NMR (300 MHz, CDCl_3_) δ: 1.23 i 1.27 (two s, 6H, =C(CH_3_)_2_), 1.55–1.63 (m, 2H, CH_2_-8), 1.61 (s, 3H, CH_3_-6), 2.00–2.08 (m, 2H, CH_2_-7), 2.11 (s, 3H, CH_3_ C(O)-), 2.21–2.28 (m, 2H, CH_2_-4), 2.44 (t, *J* = 7.4 Hz, 2H, CH_2_-3), 2.66 (t, *J* = 6.3 Hz, 1H, H-9), 5.10 (m, 1H, H-5); **^13^**C NMR (150 MHz, CDCl_3_) δ: 15.99 (C-13), 18.76 (C-12), 22.41 (C-4), 24.89 (C-11), 27.34 (C-8), 29.97 (C-1), 36.31 (C-7), 43.64 (C-3), 58.34 (C-10), 64.12 (C-9), 123.21 (C-5), 135.52 (C-6), 208.69 (C-2); IR (film, cm^−1^): 2925(s), 1718(m), 1378(m).

*(±)-9,10-epoxynerylacetone* (**4**): ^1^H NMR (300 MHz, CDCl_3_) δ: 1.07 i 1.10 (two s, 6H, =C(CH_3_)_2_), 1.39–1.43 (m, 2H, CH_2_-8), 1.50 (s, 3H, CH_3_-6), 1.93 (s, 3H, CH_3_-1), 1.96–2.01 (m, 2H, CH_2_-7), 2.04–2.11 (m, 2H, CH_2_-4), 2.27 (t, *J =* 7.4 Hz, 2H, CH_2_-3), 2.51 (t, *J* = 6.3 Hz, 1H, H-9), 4.93 (t, *J* = 7.2, 1H, H-5); ^13^C NMR (150 MHz, CDCl_3_) δ: 18.71 (C-13), 22.22 (C-4), 23.28 (C-12), 24.89 (C-11), 27.35 (C-8), 28.48 (C-7), 29.93 (C-1), 43.77 (C-3), 58.33 (C-10), 64.00 (C-9), 124.05 (C-5), 135.50 (C-6), 208.45 (C-2); IR (film cm^−1^): 2964(s), 1717(s), 1456(s), 1378(s), 1164(m).

### 4.3. General Procedures

Analytical TLC was performed on silica gel (Kieselgel 60 F_254_, Merck) with a mixture of hexane and acetone (10:1) as a developing system. Compounds were detected by spraying the plates with a solution of Ce(SO_4_)_2_ (1 g), H_3_[P(Mo_3_O_10_)_4_] (2 g) in 10% H_2_SO_4_, followed by heating to 120–200 °C.

Column chromatography was performed on silica gel (Kiesel gel 60, 230-400 mesh ASTM Merck) with a mixture of hexane:acetone 10:1 v/v as eluent.

Gas chromatography (GC) was performed on a Varian CP-3380 instrument equipped with an FID detector using an HP-1 column (cross-linked methyl silicone, 30 m × 0.53 mm × 1.5 μm). The temperature program was as follows: 110 °C (hold 1 min), 110–180 °C (rate 0.5 °C min^−1^), 180–200 °C (rate 50 °C min^−1^).

^1^H NMR and ^13^C NMR spectra were recorded in CDCl_3_ solution on a Brüker Advance DRX 300 spectrometer. IR spectra were recorded on FTIR Thermo-Mattson IR 300 Spectrometer.

### 4.4. Aphid and Plant Cultures

The laboratory culture of the green peach aphid *Myzus persicae* (Sulz.) was maintained on Chinese cabbage *Brassica rapa* L. ssp. *pekinensis* L. Aphids and plants were kept in the laboratory at 20 °C, 65% relative humidity, and L16:8D photoperiod. Five- to seven-day-old adult apterous females of *M. persicae* and three-week-old plants with four to five fully developed leaves were used for all experiments. All experiments were carried out under the same conditions of temperature, relative humidity, and photoperiod. The bioassays were started at 10:00–11:00 a.m. MEST (Middle European Summer Time). Joschinski et al. [79] found that aphids have diurnal rhythms even on constant food sources and are more active during the day than during the night.

### 4.5. Aphid Settling (Choice-Test)

The choice test allows the identification of bioactive compounds, for example, the deterrent ones [80,81]. This bioassay allows for studying aphid host preferences under semi-natural conditions. Aphids settle on a plant only when they accept it as a food source [82]. Leaves cut from cabbage plants, were dipped for 10 s in the 0.1% solution of a studied compound dissolved in 70% ethanol or control solution (70% ethanol) and dried in the air for 1 h at room temperature. Two leaves (treated and control) were transferred to Petri dishes. Afterward, 20 apterous females of *M. persicae* were placed between the leaves at the center of the Petri dish. Aphids were offered a choice between treated (on one half of a Petri dish) and control leaves (on the other half of the dish). Aphids that settled, i.e., they did not move and the position of their antennae indicated feeding [83], on each leaf were counted at 1, 2, and 24 h intervals after the beginning of the experiment. This experiment was replicated 8 times for each treatment (eight replicates, 20 viviparous apterous females/replicate). The number of aphids that settled on treated leaves was compared to the number of aphids that settled on control leaves 1, 2, and 24 h after treatment, separately for each time point, using the Student *t*-test (STATISTICA 6.1. package). If aphids showed a clear preference for the leaf treated with the tested compound (*p* < 0.05), the compound was described as having attractant properties. If aphids settled mainly on the control leaf (*p* < 0.05), the compound tested in the respective choice test was stated as a deterrent. From the data thus obtained, the relative index of deterrence (DI) was calculated: DI = (C – T)/(C + T) where C was the number of aphids settled on the control leaf, T was the number of aphids settled on the leaf treated with the tested compound. The value of DI may range from “−1” (indicating a good attractant) to “1” (indicating a good deterrent).

### 4.6. Electronic Registration of Aphid Probing Behavior (No-Choice Test)

Aphid probing behavior was monitored using the technique of electronic registration of aphid probing in plant tissues, known as EPG [84]. By using the EPG technique, it is possible to monitor aphid probing and feeding behavior within plant tissues, localize natural plant resistance factors, and reveal the effect of exogenously applied compounds that may influence plant–aphid interactions [63,85,86,87]. The EPG technique allows a separate analysis of aphid behavior at pre-ingestive (within non-phloem tissues before the first phloem phase) and ingestive (within the phloem) phases of probing [88]. The basic principle of this technique is that the aphid and plant are made parts of an electric circuit, which is completed when the aphid inserts its stylets into the plant. Weak voltage is supplied in the circuit, and all changing electric properties are recorded as EPG waveforms that can be correlated with aphid activities and stylet position in plant tissues [89]. The parameters describing aphid behavior during probing and feeding, such as the total time of probing, the proportion of activities in the phloem, the number of probes, etc., are good indicators of plant suitability or interference of probing by chemical or physical factors in plant tissues [90]. In our study, an aphid was attached to a golden wire electrode (1.5–2.0 cm long, 0.18 μm diam.) with conductive water-based silver paint (EPG-Systems, Dillenburg 126,703 CJ Wageningen, The Netherlands) and starved for 1 h prior to the experiment. The probing behavior of apterous females was monitored for 8 h continuously with the eight-channel DC EPG recording equipment to obtain 15 complete replicates. The incomplete (i.e., shorter than 8 h) recordings were excluded from the analysis. Each aphid was given access to a freshly prepared plant: one leaf of a plant was covered with the studied compound or a solvent (control). The leaf was dipped for 10 s in the 0.1% solution of the studied compound dissolved in 70% ethanol or control solution (70% ethanol) and dried in the air for 1 h at room temperature. Each aphid-plant combination was used only once and was considered one replication. The plant electrode was placed in the soil. The length of the golden wire electrode and the position of the EPG probe during the experiment (aphid + electrode + pre-amplifier) were adjusted to prevent the aphid contact with the untreated parts of the plant. Signals were saved and analyzed using the PROBE 3.1 software provided by W. F. Tjallingii (EPG-Systems, Dillenburg 126,703 CJ Wageningen, The Netherlands). The following aphid behaviors were distinguished: non-probing (waveform ‘np’–aphid stylets outside the plant), penetration of non-phloem tissues (pathway phase ‘C’ and derailed stylet movements ‘F’), phloem phase salivation into sieve elements (waveform ‘E1′), phloem phase ingestion of phloem sap (waveform ‘E2′), and xylem phase (ingestion of xylem sap, waveform ‘G’). Waveforms F and G occurred rarely, therefore were analyzed with waveform C as non-phloem phase probing activities. Waveform patterns that were not terminated before the end of the experimental period (8 h) (i.e., were artificially short due to the end of the 8 h recording) were included in the calculations. Additionally, the frequency and duration of cell punctures during pathway probing in non-phloem tissues were analyzed. These cell punctures serve as an opportunity to collect samples of cytoplasm for gustatory purposes during host plant suitability assessment by aphids [58]. Accidentally, during these cell punctures, the transmission of non-persistent and semi-persistent plant viruses may take place. The cell punctures, manifested in EPG recordings as potential drops (‘pd’) are divided into ‘short’ pds (pd-S) and ‘long’ pds (pd-L), which differ mainly in the number of pulses within the subphase II-3 that is associated with virus acquisition [61]. The cell punctures pd-S have 0–3 pulses in subphase II-3, while pd-L punctures—more than three pulses [60,61,63]. The parameters derived from EPGs were analyzed according to their frequency and duration in configuration related to activities in non-phloem and phloem tissues. All EPG parameters describing aphid probing behavior were calculated manually and individually for every aphid using the EPG analysis Excel worksheet created for this study by one of the authors (Anna Wróblewska-Kurdyk). Subsequently, the mean and standard errors were determined. Aphid behavior on leaves treated with sesquiterpenoids was compared to aphid behavior on control plants. Mann–Whitney U-test was used for these comparisons. All statistical calculations were performed using StatSoft, STATISTICA 6.1.

## 5. Conclusions

Considering the activity determined in the EPG experiments and the potency and durability of the effects determined in the aphid settling test, the compounds studied can be defined as moderate deterrents of a postingstive nature. The derivatives resulting from the epoxidation of natural compounds had a similar effect on aphid behavior as the starting compounds. The application of geranylacetone (**1**), nerylacetone (**3**), and their epoxy derivatives (**2, 4**) did not affect aphid behavior during the pre-pholem and phloem phases of stylet penetration in a significant way. However, aphids refused to settle on leaves treated with these compounds as soon as 2 h until at least 24 h after treatment. This means that the phloem sap consumed by aphids might have had toxic or deterrent properties, which made the free-moving aphids give up the treated leaves and search for an alternative food source. Nevertheless, the postingestive effect of these compounds will require a separate study into their influence on digestive physiology and/or metabolism in aphids. In addition, it is also worth extending the research on the antifeedant effects of geranylacetone and nerylacetone to rule out their lethal and sublethal effects on non-target species, e.g., predators, parasitoids, pollinators, and soil invertebrates [91].

## Figures and Tables

**Table 1:** Effect of geranylacetone (**1**), epoxygeranylacetone (**2**), nerylacetone (**3**), epoxynerolacetone (**4**) on settling of *Myzus persicae* on *Brassica rapa* subsp. *pekinensis*.

Compounds		Mean Number of Aphids		
		1 h	2 h	24 h
Geranylacetone (**1**)	test	4.6 ± 0.9	4.8 ± 0.7	2.1 ± 0.6
	control	13.5 ± 1.0	12.5 ± 1.0	10.8 ± 1.5
	*p*	0.0000	0.0000	0.0001
Epoxygeranylacetone (**2**)	test	1.8 ± 0.4	0.9 ± 0.3	1.8 ± 0.8
	control	6.9 ± 0.6	5.9 ± 1.2	5.4 ± 1.1
	*p*	0.0000	0.0012	0.0148
Nerylacetone (**3**)	test	3.9 ± 2.3	4.3 ± 2.2	2.7 ± 2.7
	control	13.4 ± 1.4	11.4 ± 0.7	12.3 ± 1.9
	*p*	0.0000	0.0000	0.0004
Epoxynerylacetone (**4**)	test	3.4 ± 0.4	4.0 ± 0.7	4.3 ± 0.6
	control	12.4 ± 0.8	6.8 ± 1.0	9.1 ± 1.5
	*p*	0.0000	0.0388	0.0093

Numbers represent the mean number of aphids (± SD) that settled on the treated (test) and control leaves. Student *t*-test at *p* = 0.05 was used to compare the number of aphids on treated and control leaves at each time point, separately.

**Table 2:** Probing behavior (EPG parameters) of *Myzus persicae* on *Brassica rapa* subsp. *pekinensis* leaves treated with geranylacetone (**1**), epoxygeranylacetone (**2**), nerylacetone (**3**), and epoxynerylacetone (**4**) ^A^. The same plant was used to maintain the aphid stock culture.

EPG Parameter	Compounds				
	Control	1	2	3	4
	**General Aspects of Aphid Probing Behavior**				
	n = 15	n = 15	n = 15	n = 15	n = 15
Total duration of probing ^a^ (h)	7.0 ± 1.3	6.9 ± 1.1	6.7 ± 1.7	7.2 ± 0.8	7.5 ± 0.3 *
Total duration of probing in non-phloem tissues ^b^ (h)	4.1 ± 2.1	3.5 ± 2.1	3.0 ± 2.0	4.0 ± 2.2	3.9 ± 2.4
Total duration of phloem phase ^c^ (h)	2.9 ± 2.3	3.4 ± 2.6	3.8 ± 2.3	3.2 ± 2.7	3.7 ± 2.7
Total number of probes ^d^ (#)	24.7 ± 9.8	27.9 ± 20.9	29.0 ± 18.5	23.1 ± 21.0	21.1 ± 15.1
Mean duration of a probe ^d^ (min)	21.7 ± 17.2	51.3 ± 109.5	22.9 ± 18.5	42.3 ± 35.8	47.5 ± 52.6
Duration of first probe ^d^ (min)	1.8 ± 4.3	33.0 ± 113.1	3.2 ± 6.8	14.1 ± 30.2	6.6 ± 21.2
Number of probes with phloem phase ^d^ (#)	1.9 ± 1.0	1.3 ± 0.6	1.3 ± 0.7	1.5 ± 1.0	1.7 ±1.3
Proportion of aphids reaching phloem phase (#)	0.9 ± 0.3	0.9 ± 0.3	0.9 ± 0.3	0.9 ± 0.4	0.9 ± 0.4
Time from first probe to first phloem phase (h)	2.4 ± 2.1	3.9 ± 2.9	3.1 ± 2.3	3.3 ± 2.7	2.1 ± 2.5
Number of phloem phases (#)	4.7 ± 3.0	1.7 ± 1.2 *	2.0 ± 1.7 *	3.0 ± 2.6	3.3 ± 3.7
Number of sustained sap ingestion phases ^e^ (#)	2.1 ± 1.7	1.2 ± 0.9	1.5 ±1.1	1.8 ± 1.8	1.4 ± 1.2
Phloem phase index ^f^ (#)	0.4 ± 0.3	0.5 ± 0.3	0.5 ± 0.3	0.4 ± 0.3	0.5 ± 0.3
	**Activities in Non-Phloem Tissues before the First Phloem Phase**				
	n = 14	n = 14	n = 14	n = 13	n = 13
Total duration of non-probing before first phloem phase ^B^ [h]	0.4 ± 0.3	1.0 ± 1,1	0.6 ± 0.5	0.3 ± 0.4	0.2 ± 0.2
Total duration of probing in non-phloem tissues before first phloem phase ^B^ (h)	1.6 ± 1.3	2.7 ± 2.0	2.1 ± 1.5	2.2 ± 1.8	1.1 ± 0.7
Number of probes before first phloem phase ^B^ (#)	13.2 ± 9.6	23.6 ± 23.1	20.0 ± 18.2	12.1 ± 14.1	7.7 ± 8.4
Number of probes < 3 min. before first phloem phase ^B^ (#)	8.0 ± 6.5	15.1 ± 16.6	14.2 ± 14.3	7.1 ± 9.3	4.1 ± 5.4
	**Activities in Sieve Elements**				
	n = 14	n = 14	n = 14	n = 13	n = 13
Duration of first phloem phase ^B^ (h)	0.6 ± 1.5	1.8 ± 2.4	2.3 ± 2.3	1.3 ± 1.9	3.0 ± 3.0
Mean duration of sap ingestion ^B^ (h)	0.9 ± 1.4	2.3 ± 2.2 *	2.1 ± 2.0	1.5 ± 1.9	2.8 ± 2.8 *
Phloem salivation index ^g^ (#)	0.1 ± 0.1	0.02 ± 0.05	0.1 ± 0.2	0.1 ± 0.3	0.03 ± 0.1

^A^ Values are means ± SD; n = number of replications; asterisk * denotes a significant difference in relation to control (*p* < 0.05, Mann–Whitney U test); ^B^ only aphids that showed a phloem phase were used for analysis; ^a^ period when aphid stylets were withdrawn from plant tissues; ^b^ C + G + F; ^c^ E1 + E2; ^d^ a probe includes all probing activities from the insertion of stylets into plant tissues until the withdrawal of stylets; ^e^ E2 > 10 min; ^f^ E1 + E2/(C + G + E + F); ^g^ E1/(E1 + E2). Waveforms: C—stylet pathway phase; F—stylet penetration difficulties; G—active ingestion of xylem sap; E1—phloem salivation, E2—phloem feeding. # means number.

**Table 3:** Exploratory cell punctures made by *Myzus persicae* in in non-phloem leaf tissues of *Brassica rapa* subsp. *pekinensis* after application of geranylacetone (**1**), epoxygeranylacetone (**2**), nerylacetone (**3**), epoxynerylacetone (**4**) ^A^. The same plant was used to maintain the aphid stock culture.

EPG Parameter	Compounds				
	Control	1	2	3	4
	n = 15	n = 15	n = 15	n = 15	n = 15
Proportion of probes with potential drops pd-S and pd-L (%)	80.1 ± 11.5	67.0 ± 21.9	62.1 ± 19.8 *	72.2 ± 19.4	77.4 ± 12.4
**Short Potential Drops (pd-S)**					
Total number of pd-S (#)	126.7 ± 64.8	95.0 ± 44.1	84.3 ± 46.1 *	100.7 ± 45.2	116.4 ± 74.5
Number of pd-S during a single penetration (#)	7.0 ± 2.8	9.1 ± 10.3	6.4 ± 3.9	10.4 ± 6.2	10.1 ± 6.9
Mean duration of a single pd-S (s)	5.1 ± 1.6	4.3 ± 0.4 *	5.7 ± 2.1	4.2 ± 1.2 *	4.7 ± 0.9
Total duration of subphase II-1 of pd-S (s)	301.1 ± 229.0	175.2 ± 78.9 *	465.6 ± 370.9	174.7 ± 77.8 *	237.1 ±145.5
Total duration of subphase II-2 of pd-S (s)	146.4 ± 72.1	93.0 ± 40.3 *	95.7 ± 84.0 *	97.1 ± 43.8 *	122.3 ± 80.6
Total duration of subphase II-3 of pd-S (s)	209.4 ± 128.2	140.4 ± 70.5	220.1 ± 200.3	157.3 ± 147.2	190.0 ± 128.1
**Long Potential Drops (pd-L)**					
Total number of pd-L (#)	6.3 ± 4.2	6.6 ± 6.8	4.3 ± 4.4	5.8 ± 5.7	9.4 ± 8.2
Number of pd-L during a single penetration (#)	0.3 ± 0.2	0.6 ± 0.7	0.2 ± 0.2	0.5 ± 0.3	0.7 ± 0.4 *
Mean duration of a single pd-L (s)	6.4 ± 2.2	5.7 ± 2.4	5.9 ± 2.5	5.8 ± 0.7 *	6.6 ± 1.1
Total duration of subphase II-1 of pd-L (s)	10.7 ± 7.4	11.2 ±10.8	7.1 ± 6.9	9.4 ± 10.2	17.6 ± 16.6
Total duration of subphase II-2 of pd-L (s)	7.1 ± 4.9	6.6 ± 6.5	4.9 ± 4.7	5.9 ± 5.7	10.1 ± 9.3
Total duration of subphase II-3 of pd-L (s)	26.9 ± 23.1	25.1 ± 27.3	17.8 ± 19.1	17.5 ± 14.6	37.5 ± 39.9

^A^ Values are means ± SD; n = number of replications; * significant difference in relation to control (*p* < 0.05, Mann–Whitney U test). # means number.

## Data Availability

Data used for this study are available from the Authors at request.

## Sample Availability

Samples of the compounds are available from the authors.
